# Development and Validation of a Clinical Prediction Rule to Identify Preoperatively Patients With Acute Refractory Pain After Lumbar Spine Surgery

**DOI:** 10.1111/papr.70213

**Published:** 2026-10-14

**Authors:** Ilse H. van de Wijgert, Kris C. P. Vissers, Martijn W. Heymans, Akkie Rood, Moniek de Goeij, Regina L. M. van Boekel, Miranda L. van Hooff

**Affiliations:** ^1^ Department of Anesthesiology Sint Maartenskliniek Nijmegen the Netherlands; ^2^ Department of Anesthesiology, Pain and Palliative Medicine Radboud University Medical Center Nijmegen the Netherlands; ^3^ Department of Research Sint Maartenskliniek Nijmegen the Netherlands; ^4^ Department of Epidemiology and Biostatistics VU University Medical Center Amsterdam the Netherlands; ^5^ Department of Orthopedic Surgery Sint Maartenskliniek Nijmegen the Netherlands; ^6^ Research Department of Emergency and Critical Care HAN University of Applied Sciences Nijmegen the Netherlands; ^7^ Department of Orthopedic Surgery Radboud University Medical Center Nijmegen the Netherlands

## Abstract

**Background:**

Preoperative risk stratification to identify patients who develop acute refractory pain after lumbar spine surgery is a first step to prevent unfavorable outcomes. In this study, we aimed to develop and internally validate a clinically relevant, multidimensional prediction rule for preoperative risk assessment.

**Methods:**

This is a retrospective analysis of prospectively collected data conducted at an orthopedic hospital specialized in spine care. Baseline and outcome data on patients who underwent lumbar spine surgery between July 2012 and December 2022 were included in this study. 105 factors were considered as potential predictive factors for acute refractory pain after lumbar spine surgery. Multivariable logistic lasso regression analysis and multivariable logistic regression analysis were performed to develop a prediction model. Performance measures (ROC‐AUC and Nagelkerke's *R* ^2^) and a prediction rule for clinical use were presented.

**Results:**

Data on 1156 patients was included in this analysis. The prevalence of patients developing acute refractory pain was 19.6% (*n* = 228/1156). Multivariable logistic lasso regression identified 13 relevant predictive factors for acute refractory pain after lumbar spine surgery: age, prior hospital treatment for back pain, professional sports participation, duration of back pain, back pain before age 45, numeric pain rating scale for back pain, preoperative opioid use, circumferent surgery, instrumented surgery, anxiety, catastrophizing, return‐to‐work attitude, high score for psychosocial burden of low back pain. Five of these were selected by logistic regression. The discriminative ability of the multivariable logistic lasso regression model was ROC‐AUC of 0.69 (95% CI 0.66–0.73), and the multivariable logistic regression model 0.67 (95% CI 0.63–0.71). Nagelkerke's *R* ^2^ of the logistic regression model was 8.4%.

**Conclusion:**

Multidimensional predictive factors for acute refractory pain were identified using a clinical prediction rule. This rule creates a patient profile which can help anesthesiologists to preoperatively determine the individual risk for developing acute refractory pain despite the use of a standardized multimodal analgesic treatment.

## Highlights

- Nearly one in five patients (19.8%) developed acute refractory pain despite standardized multimodal analgesia after lumbar spine surgery.
- Thirteen multidimensional predictors of acute refractory pain were identified and incorporated into a clinically applicable prediction rule, including demographic, clinical, surgical, and psychosocial factors.
- Preoperative risk stratification may support individualized perioperative pain management and shared decision‐making, and may facilitate tailored interventions for modifiable risk factors.

## 1 Introduction

To date, acute postoperative pain is present in more than 50% of patients undergoing spinal fusion surgery [1]. This type of surgery is deemed the second and third most painful surgery when compared to 179 other surgical procedures [2]. Acute pain causes immobility, accompanied by postoperative complications, prolonged hospital admission. It can result in acute‐on‐chronic postsurgical pain with concomitant loss of quality of life [3, 4, 5, 6]. It is of pivotal importance to achieve better pain control in lumbar spine surgery, as the prevention of pain is better than any treatment of pain.

Strategies to prevent pain are widely studied, such as the implementation of Enhanced Recovery After Surgery (ERAS) programs. These programs describe the use of a multimodal analgesic approach, including the use of opioids to achieve acceptable postoperative pain [7, 8, 9]. The use of postoperative opioids is associated with chronic opioid use [10], which rises a challenge particularly important in patients undergoing spine surgery. Many of these patients preoperatively suffer from chronic low back and leg pain, have often undergone multiple surgeries, and are extensively exposed to long‐term opioid administration causing opioid dependence and tolerance [11]. Postoperative pain treatment as described in the ERAS programs are then no longer sufficient, resulting in acute refractory pain. This results in the need for so‐called rescue analgesics (i.e., esketamine, clonidine, benzodiazepines) to comfort patients suffering from acute refractory pain. Hence, the preoperative identification of those patients should help to prevent this pain by using individualized, appropriate treatment strategies.

Preoperative identification can be achieved using patient characteristics. Patient characteristics that predict acute postoperative pain are widely studied. Following their review, Yang et al. [11, 12] developed a clinical prediction model for identification of patients undergoing lumbar spine surgery who are at‐risk for inadequate pain control. Although this model is of great potential as a clinically relevant and applicable prediction model, up to 38% of patients are still misclassified. This implies that other factors influence the outcome, but those were undetected. For example, psychosocial factors have been proven to play a role in the occurrence of acute postoperative pain, such as anxiety, catastrophizing, uncertainty that the operation will succeed, and thoughts of undergoing a dangerous surgical procedure [12, 13, 14, 15]. Combining a wide range of biopsychosocial characteristics in a clinical prediction model can enhance patient care by improved identification preoperatively of patients who are at risk of developing postoperative refractory pain.

In this study, we aimed to determine first the prevalence of acute refractory pain after lumbar spine surgery. And second, the goal was to develop and internally validate a clinically relevant, multidimensional clinical prediction model. This model was then converted to a clinical prediction rule that creates patient profiles which can help to determine preoperatively an individual's risk of developing acute refractory pain despite the use of multimodal analgesic treatment modalities in patients for spinal fusion surgery.

## 2 Methods

### 2.1 Study Design

This is a retrospective analysis of prospectively collected data conducted at an orthopedic hospital specialized in spine care (Sint Maartenskliniek, Nijmegen, the Netherlands). The study follows the guidelines of the *Transparent reporting of a multivariable prediction model for individual prognosis or diagnosis* (TRIPOD) statement [16]. All analyses were performed in *Rstudio*, version 4.1.3 (2022). The R‐code was made available for transparency via Github along with publication of this article in File S3.

### 2.2 Source of Data

In the Sint Maartenskliniek (Nijmegen, the Netherlands), all consecutive referred low back pain patients receive a web‐based screening questionnaire including baseline patient‐reported outcome measures (PROMs). This is part of a patient outcomes spine registry and decision support (the Nijmegen Decision‐support Tool for Chronic Low Back Pain; NDT‐CLBP) [13]. The tool is used to support preoperative patient triage and has been implemented in routine practice since 2012 [13, 17]. Data from patients who completed this questionnaire and subsequently underwent lumbar spine surgery were included in this study. Although originally developed to support treatment selection in patients with low back pain, the registry includes a broad range of preoperative biopsychosocial variables that are also plausible determinants of acute postoperative pain outcomes. Preoperative registry data were matched to the electronic medical record (EMR) data using the operation number. The EMR data contains outcome data on pain intensity, use of rescue analgesics, and other clinically relevant parameters collected in the post‐anaesthesia care unit (PACU) until discharge to the medical ward. Data was pseudonymized directly after merging, and the encryption key was stored separately.

### 2.3 Participants

Data on patients who underwent elective lumbar spine surgery between July 2012 and December 2022 were included. Other inclusion criteria were the presence of EMR data and age ≥ 16 years. Exclusion criteria were: no completed pre‐treatment NDT‐CLBP screening questionnaire available, no operation date, the questionnaire did not relate to the index operation, no outcome measurement available, surgery because of trauma, malignancy, or adolescent scoliosis (receiving intrathecal/epidural anesthesia), emergency surgery, and patients with an American Society of Anesthesiologists (ASA) score of ≥ 4.

### 2.4 Primary Outcome Measure

The primary outcome was acute refractory pain (dichotomized to yes [‘refractory pain’]/no [‘manageable pain’]) during PACU stay despite the use of a standardized multimodal pain treatment protocol (Table 1).

**TABLE 1:** Stepwise standardized multimodal treatment protocol for lumbar spine surgery.

Treatment phase	Medication
Preoperative	Midazolam
	Gabapentin
	Paracetamol
	Etoricoxib
Induction	Dexamethasone
	Sufentanil
	Lidocaine
	Propofol
	Esketamine
	Rocuronium
Perioperative	Sevoflurane or propofol
	Sufentanil or remifentanil
	Morphine or piritramide
	Ondansetron
Postoperative	Paracetamol
	Etoricoxib
	Morphine or piritramide
Postoperative rescue analgesics	Clonidine
	Esketamine
	Benzodiazepines (diazepam)

Acute refractory pain was defined using the following criteria:

- Reporting of maximum NRS ≥ 7 on the PACU *AND* at least one of the following criteria:
  ○ Acute consultation of anesthesiologist for pain management and action taken in response to this consultation, *OR*;
  ○ Use of rescue analgesics during PACU stay (Table 1), *OR*;
  ○ Overnight PACU stay because of uncontrollable pain, *OR*;
  ○ Reporting of apnea (indicating ‘too much morphine necessary for pain control’).

A pilot on this composite outcome measure was conducted prior to final data collection to assess the feasibility of the use of this outcome measure, using postoperative data on patients operated from January to December 2019 (*n* = 363). Analyses showed it was feasible to obtain the defined primary outcome for all patients included. The prevalence of acute refractory pain in this pilot‐population was 20.7% (*n* = 75/363). More details on this pilot procedure are available in File S1.

### 2.5 Potential Predictive Factors

Factors as measured by the NDT‐CLBP were used as potential predictive factors for the development of the prediction model [13]. These factors covered five domains (i.e., socio‐demographic, pain, somatic, psychological, and functioning/quality of life); an overview of the 105 unique potential predictive factors, as well as their ranges of values/categories, are displayed in File S2. We performed data inspection of every potential predictive variable. Descriptive statistics of these factors were obtained. A factor was excluded in case of > 5% missing values or when no events for the outcome were present. The predictive factors were assessed for collinearity using the variance inflation factor (VIF). VIF values > 10 were considered a sign for a high, intolerable correlation of model predictors [18]. Continuous potential predictive factors were checked for having a linear relationship with the outcome using restricted cubic spline regression [19]. In case of a non‐linear relationship, spline coefficients were applied in logistic regression.

### 2.6 Statistical Analysis

At first, central tendency measures of the factors were summarized to describe the distribution and value of the factors. Mean (standard deviation) and median (interquartile range) were used for continuous variables depending the presence of a normal distribution. For nominal variables relative frequencies (counts [percentages]) were calculated. The prevalence of acute refractory pain was calculated.

#### 2.6.1 Variable Selection for Model Development Using Multivariable Logistic Lasso Regression

A complete‐case analysis including all potential predictive factors was performed when the percentage of patients with missing data was less than 5%, and the missing data were analyzed for randomness. Variable selection was performed using multivariable logistic lasso (least absolute shrinkage and selection operator) regression. A suitable value of Lambda was determined by cross‐validation. The selected predictive factors were presented in a Clinical Prediction Rule displaying the (shrunk) regression coefficients. No inferential conclusions can be drawn because the regression coefficients are shrunk. However, to provide an effect estimate of predictors, variables selected by lasso were put together in a normal multivariable logistic regression to obtain adjusted odds ratios (aOR) with their 95% confidence intervals (95% CI).

### 2.7 Performance of the Prediction Model

The model's discriminative ability was explored by creating a ‘receiver operating characteristics’ (ROC) curve. The ROC‐area under the curve (ROC‐AUC), that is, C‐statistics with 95% CI, was determined.

### 2.8 Clinical Prediction Rule

The general structure of a prediction rule, presented as a logistic regression model, is based on a linear combination of predictive factors. The equation looks like this:

$$y=\beta_{0}+\beta_{1}\times {\text{predictor}}_{1}+\beta_{2}\times {\text{predictor}}_{2}+\beta_{\kappa }\times {\text{predictor}}_{\kappa }+\epsilon$$

In which y = the primary outcome (acute refractory pain); $\beta_{0}$ = intercept; $\beta_{1},\beta_{2},\ldots ,\beta_{\kappa }$ = coefficients for each corresponding determinant (predictive factor); ${\text{predictor}}_{1},{\text{predictor}}_{2},\ldots ,{\text{predictor}}_{\kappa }$ = independent variables (predictive factors); ϵ = error term.

The prediction rule resulting from this study's multivariable logistic lasso regression analyses was obtained by using the lasso coefficients of the relevant predictive factors. To convert the linear combination y to individual predicted probabilities (p), the following function was used:

$$p=\frac{1}{1+e^{-y}}$$

#### 2.8.1 Clinical Utility

To make the prediction model applicable in clinical practice, a cut‐off value for predicted probability of developing acute refractory pain was determined using the Index of Union. This is the “optimal” cut‐off point which classified most of the individuals correctly and thus least of them incorrectly [20]. A high specificity was favorable above a high sensitivity because the goal is to identify patients with elevated risk of acute refractory pain despite using a multimodal analgesic protocol. The cut‐off value was used for risk stratification into high or low risk of developing acute refractory pain.

By means of exploratory impact‐analysis, the net benefit of the prediction rule was compared with the default policy by using a decision curve analysis [21]. The default policy is to operate everyone with an indication to undergo spine surgery with absence of significant comorbidity or contra‐indications. By implementing the previously established cut‐off value for risk stratification, the accuracy between the default policy and the accuracy of the prediction rule is defined as the net benefit that can be achieved in clinical practice.

### 2.9 Additional Analyses

#### 2.9.1 Variable Selection Using Multivariable Logistic Regression

For transparency, multivariable logistic regression analysis without lasso was performed to assess differences in factor selection between the lasso and logistic regression selection methods. In the latter, a selection of potential predictive factors was made to create a parsimonious model. The potential predictive factors were rated on several characteristics: clinical relevance, relevance in literature, missing values, prevalence, and duplicates. The institution's anesthesiologists were invited to rate the predictive factors on clinical relevance. A prediction model was developed and internally validated using multivariable logistic regression analysis with backward selection and bootstrapping with 250 iterations using the selected list of factors. Factors with the highest *p* value were removed when *p* > 0.05, until all remaining factors had a *p* < 0.05. Optimism in the model's regression coefficients, due to overfitting, was estimated by the calibration slope. A slope of 1.0 indicates no optimism by overfitting. The model was adjusted by shrinkage of the coefficients and adjustment of the intercept. These adjusted coefficients and intercept were used to obtain a prediction rule. Performance of the model was reported by means of the ROC‐AUC and the percentage of variance explained (Nagelkerke's) *R* ^2^. The performance of the final multivariable logistic lasso regression model was compared to the performance of the logistic regression model.

#### 2.9.2 Univariable Analysis of Selected Predictive Factors

Lastly, univariable analysis of the selected predictive factors was performed to show the crude relationship between the predictive factor and the outcome measure.

#### 2.9.3 Sensitivity Analysis

Sensitivity analysis was performed to identify profiles for patients at risk for developing severe acute postoperative pain, that is, a NRS of ≥ 4. This was performed to enhance comparability with other research into predictive factors for severe postoperative pain.

## 3 Results

### 3.1 Participants

Web‐based screening questionnaires were completed from 02‐03‐2012 until 31‐12‐2022 by 6951 patients undergoing spine surgery. Of those, 1404 were applicable to merge with outcome data. Parallelly, data of 5401 patients were derived from EMR. Of those, 4690 were usable to merge with the spine registry data. After merging on operation number, data of 4888 patients remained. Finally, data from 1156 patients were included in analysis. A data flow diagram of this study is provided in Figure 1.

**FIGURE 1:**
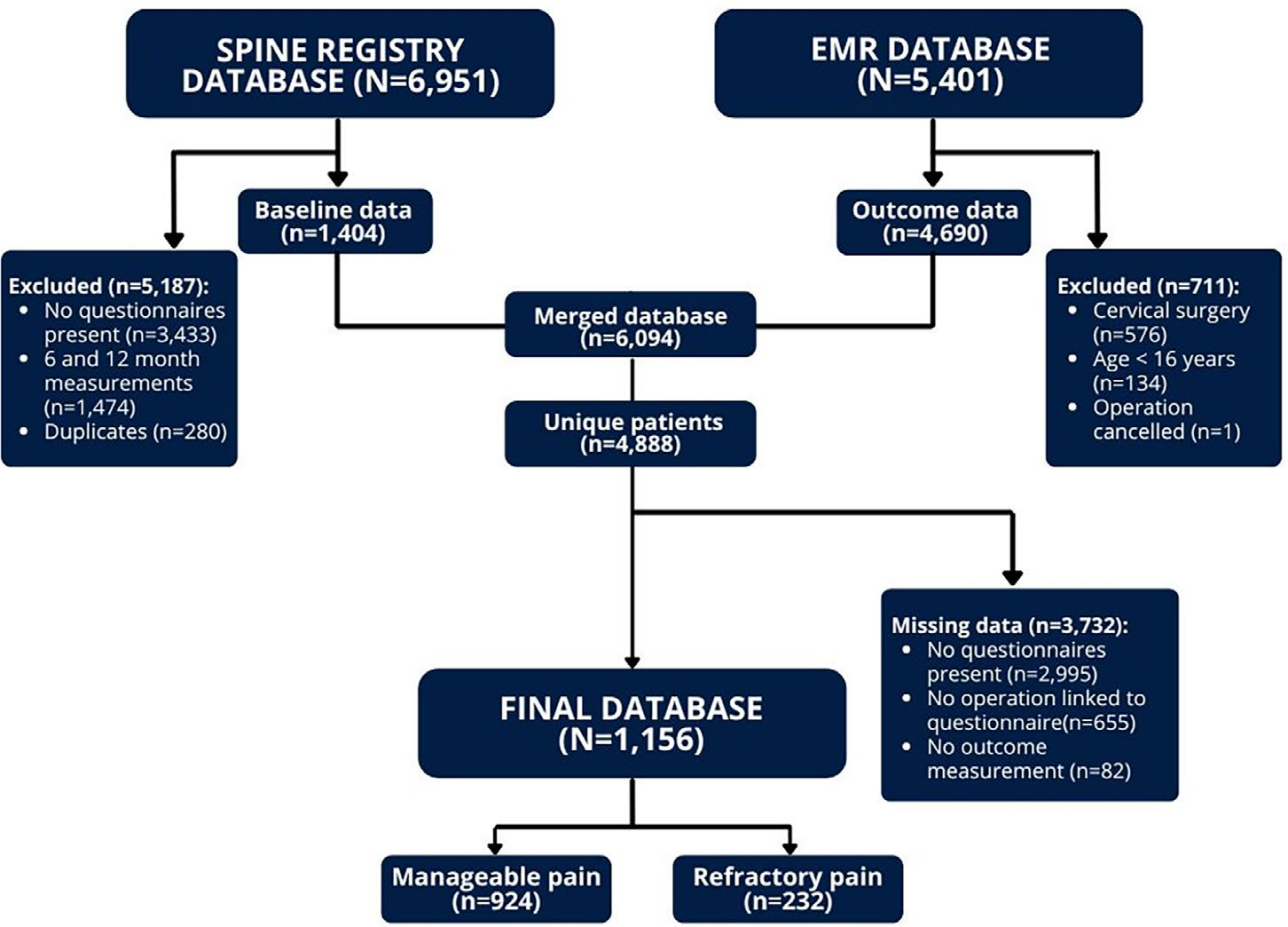
Data flow chart. EMR, electronic medical record.

### 3.2 Primary Outcome Measure

The prevalence of acute refractory pain was 19.7% (*n* = 228/1156). This was similar to the pilot outcome measurement (20.7%, *n* = 75/363). In Table 2 the patients' demographic characteristics are presented.

**TABLE 2:** Demographics refractory pain versus manageable pain.

Variable	Refractory pain (*n* = 228)	Missing values (*n*)	Manageable pain (*n* = 928)	Missing values (*n*)
Sex [*n*, female (%)]	138 (60.5%)	0	561 (60.5%)	0
Age in years (median, IQR)	54 (45–65)	0	59 (49–69)	0
BMI in kg/m^2^ (mean, SD)	26.8 (±3.9)	11	26.9 (±4.0)	30
Smoking [*n*, yes (%)]	43 (18.9%)	0	167 (18%)	0
ASA‐score (*n*, %)		4		13
ASA 1	63 (27.6%)		248 (26.7%)	
ASA 2	149 (65.4%)		608 (65.5%)	
ASA 3	12 (5.3%)		59 (6.4%)	
Planned surgery (*n*, %)		0		0
Single level instrumented	130 (57.0%)		444 (47.8%)	
Multi‐level instrumented	32 (14.0%)		94 (10.1)	
Un‐instrumented	66 (28.9%)		390 (42%)	
CLBP^a^ [*n*, yes (%)]	219 (96.0%)	0	876 (94.4)	0
Duration CLBP [*n* (%)]		0		0
< 2 years	27 (11.8%)		177 (19.1%)	
2–5 years	71 (31.1%)		315 (33.9%)	
6–10 years	46 (20.2%)		132 (14.2%)	
> 10 years	84 (36.8%)		304 (32.8%)	
Radiculopathy [*n*, yes (%)]	203 (89.0%)	0	826 (89.1%)	1
Preoperative NRS back pain (mean ± SD)	7.3 (±1.9)	0	6.8 (±2.2)	0
Preoperative NRS leg pain (mean ± SD)	6.3 (±2.6)	0	6.5 (±2.7)	0
Preoperative opioid use [*n*, yes (%)]	112 (49.1%)	0	305 (32.9%)	0
Preoperative ODI (mean ± SD)	47.5 (±16.3)	0	44.4 (±15.0)	0
Preoperative EQ‐5D (mean ± SD)	0.4 (±0.3)	0	0.5 (±0.3)	0
Preoperative EQ‐VAS (mean ± SD)	53.2 (±21.3)	1	56.1 (±20.5)	2
Previous back surgery [*n*, yes (%)]	103 (46.4%)	0	376 (41.5%)	0
SBST risk profile (*n*, %)		0		0
Low risk	28 (12.3%)		164 (17.7%)	
Medium risk	164 (71.9%)		669 (72.1%)	
High risk	36 (15.8%)		95 (10.2%)	

Abbreviations: CLBP, chronic low back pain; EQ‐5D, EuroQol 5 Dimensions; EQ‐VAS, EuroQol Visual Analogue Scale; IQR, interquartile range; NRS, numeric rating scale; ODI, Oswestry Disability Index; SBST, Start Back Screening Tool; SD, standard deviation.

^a^ Longer than 3 months.

### 3.3 Predictive Factors

Of the initial list of 105 potential predictive factors, 19 were excluded due to having > 5% missing values. Furthermore, 25 factors had no events for the outcome, which led to exclusion of those. Thus, 61 factors could be included in the multivariable logistic lasso regression analysis.

For the additional multivariable logistic regression analysis, a parsimonious model was established by selecting a total of 27 unique potential predictive factors. This number was achieved because of the 61 factors included in the multivariable logistic lasso regression, 21 were not deemed clinically relevant. Two potential factors were not associated with pain or opioid use in scientific literature. Lastly, 11 potential factors were excluded because these factors measured the same constructs as some other factors did (i.e., duplicates). All potential and selected predictive factors used in multivariable logistic regression analysis are shown in File S2. No multicollinearity was found among those factors. The continuous factors were assessed for linearity using the restricted cubic spline function. No assumptions were violated.

### 3.4 Development of the Prediction Model

#### 3.4.1 Variable Selection for Model Development Using Multivariable Logistic Lasso Regression

As the percentage of patients with missing data was less than 5% (29/1156; 2.5%), a complete‐case analysis was deemed appropriate. Review of the missing cases indicated that missingness was attributable to administrative causes rather than patient or outcome characteristics. The multivariable logistic lasso regression analysis was performed using the remaining data of 1127 patients and 61 potential predictive factors. Cross validation led to a Lambda value (Lambda.min) of 0.02, with 15 degrees of freedom (Df), which was used to obtain the prediction model (Figure 2A,B). The range in predicted probabilities was limited, as shown in the calibration curve (Figure 2C). The model had an ROC‐AUC of 0.69 (95% CI 0.66–0.73) (Figure 2D). The multivariable logistic lasso regression model included the following factors: age, prior hospital treatment for back pain, professional sports participation, duration of back pain, back pain before age 45, numeric pain rating scale for back pain, preoperative opioid use, circumferent surgery, instrumented surgery, anxiety, catastrophizing, return‐to‐work attitude, high score for psychosocial burden of low back pain. The prediction rule using the shrinked regression coefficients are displayed in the Clinical Prediction Rule. The effect estimates (aORs; 95% CI) of selected predictive variables using a normal multivariable are presented in Table 3.

**FIGURE 2:**
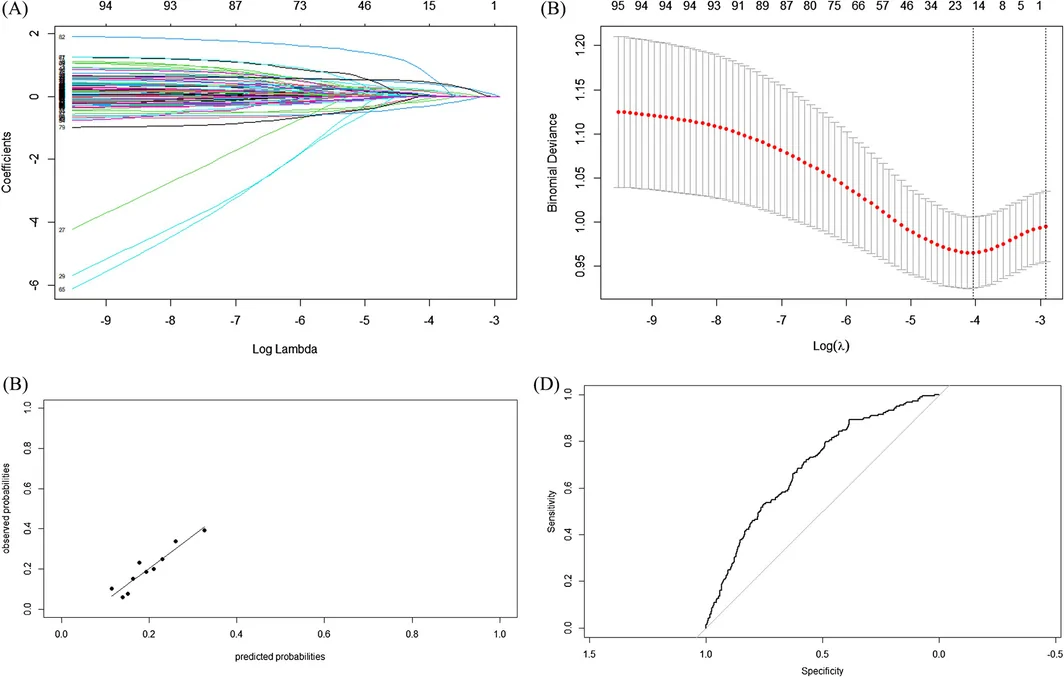
Multivariable logistic lasso regression. (A) Lasso coefficient profiles of the variables; (B) cross validation indicating Lambda.min; (C) calibration plot (dotted line indicates perfect calibration); (D) ROC‐AUC 0.69 (95% CI 0.66–0.73).

**TABLE 3:** Predictive variables selected by multivariable logistic lasso regression associated with refractory pain after spine surgery.

Domain	Parameter	aOR^a^	95% CI^a^	*p*
Socio‐demographic (Personal)	Age (years)	0.99	−0.03; 0.01	0.35
Socio‐demographic (Health)	Previous hospital treatment for back pain (No = 0; Yes = 1)	1.51	0.08; 0.74	0.08
Socio‐demographic (Social)	*Sports participation*			
	No (0)	Reference	Reference	Reference
	Professional (1)	8.04	0.18; 3.98	0.03*
	Recreational (2)	0.79	−0.57; 0.09	0.16
Pain	*Duration back pain*			
	No back pain (0)	Reference	Reference	Reference
	2–5 years (1)	1.36	−0.14; 0.76	0.19
	5–10 years (2)	1.14	−0.24; 0.50	0.50
	> 10 years (3)	0.70	−0.88; 0.18	0.19
	Back pain before age 45 (No = 0; Yes = 1)	0.58	−0.91; −0.17	< 0.01*
	Numeric Pain Rating Scale back pain (0–10)	1.04	−0.06; 0.14	0.38
	Preoperative opioid use (No = 0; Yes = 1)	1.84	0.28; 0.94	< 0.01*
Somatic	Circumference surgery (No = 0; Yes = 1)	1.44	−0.05; 0.77	0.09
	Surgery type (Un‐instrumented = 0; Instrumented = 1)	1.39	−0.04; 0.70	0.08
Psychological (Psychic affect)	*Anxiety*			
	No (0)	Reference	Reference	Reference
	Moderate (1)	0.80	−0.59; 0.15	0.23
	Very bad (2)	1.74	0.05; 1.07	0.03*
Psychological (Cognition)	Catastrophizing (No = 0; Yes = 1)	1.33	−0.04; 0.62	0.10
Psychological (Behavior)	*Return‐to‐work attitude*			
	Currently working (0)	Reference	Reference	Reference
	Fulltime return (1)	0.56	−1.18; −0.04	0.04*
	Parttime return (2)	1.06	−0.49; 0.61	0.83
	Wish to change job (3)	1.44	−0.25; 0.97	0.24
	Wish to keep paid sick leave (4)	1.08	−0.50; 0.64	0.80
	Wish to keep retirement benefit (5)	0.84	−0.69; 0.33	0.49
Functioning and quality of life	*STarT Back Screening Tool* [22]			
	Low risk (0)	Reference	Reference	Reference
	Medium risk (1)	1.22	−0.29; 0.69	0.44
	High risk (2)	1.65	−0.17; 0.17	0.14

Abbreviations: 95% CI, 95% confidence intervals; aOR, adjusted odds ratio, values of multivariable analysis. aOR > 1 means a higher risk of refractory pain and an aOR < 1 means a lower risk of refractory pain.

^a^ Values are obtained from normal multivariable logistic regression for presentation of effect estimates. These values are not shrunk and thus overestimation of effect could have occurred.

* Statistically significant with *p*‐values < α = 0.05.

#### 3.4.2 Clinical Prediction Rule

The prediction rule as adopted from the multivariable logistic lasso regression analysis was as follows:

$$\begin{array}{c} y=-1.9428\\ -0.01\times \left(\mathrm{age}\left(\text{years}\right)\right)\\ \begin{array}{c} +0.41\times \left(\text{previous hospital treatment for back pain}:\mathrm{no}=0,\mathrm{yes}=1\right)\\ +2.08\times \left(\text{sports participation}:\text{professional}\right)-0.24\times \left(\text{sports participation}:\text{recreational}\right)\\ \begin{array}{c} +0.31\times \left(\text{duration of back pain}:2-5\text{years}\right)+0.13\times \left(\text{duration of back pain}:5-10\text{years}\right)\\ -0.35\times \left(\text{duration of back pain}:>10\;\text{years}\right)\\ \begin{array}{c} -0.54\times \left(\text{back pain before}\;\mathrm{age}\;45:\mathrm{no}=0,\mathrm{yes}=1\right)\\ +0.04\times \left(\text{Numeric Pain Rating Scale for back pain}\left(0-10\right)\right)\\ \begin{array}{c} +0.61\times \left(\text{preoperative opioid}\;\mathrm{use}:\mathrm{no}=0,\mathrm{yes}=1\right)\\ +0.36\times \left(\text{circumferent surgery}:\mathrm{no}=0,\mathrm{yes}=1\right)\\ \begin{array}{c} +0.33\times \left(\text{surgery type}:\text{uninstrumented}=0,\text{instrumented}=1\right)\\ -0.22\times \left(\text{anxiety}:\text{moderate}\right)+0.56\cdot \left(\text{anxiety}:\text{very}\;\mathrm{bad}\right)\\ \begin{array}{c} +0.29\times \left(\text{catastrophizing}:\mathrm{no}=0,\mathrm{yes}=1\right)\\ -0.61\times \left(\text{return}-\text{to}-\text{work attitude}:\text{full}-\text{time return}\right)\\ \begin{array}{c} +0.06\times \left(\text{return}-\text{to}-\text{work attitude}:\text{part}-\text{time return}\right)\\ +0.36\times \left(\text{return}-\text{to}-\text{work attitude}:\text{wish to change}\;\mathrm{job}\right)\\ \begin{array}{c} +0.07\times \left(\text{return}-\text{to}-\text{work attitude}:\text{wish to keep paid sick leave}\right)\\ -0.18\times \left(\text{return}-\text{to}-\text{work attitude}:\text{wish to keep retirement benefit}\right)\\ \begin{array}{c} +0.20\times \left(\text{STarT Back Screening Tool}:\text{medium risk}\right)\\ +0.50\times \left(\text{STarT Back Screening Tool}:\text{high risk}\right) \end{array} \end{array} \end{array} \end{array} \end{array} \end{array} \end{array} \end{array} \end{array} \end{array}$$

The most optimal cut‐off was determined as described in File S4 and resulted in a probability of 22.3% for stratification of patients into low or high risk of developing acute refractory pain. This means that patients with a calculated predicted probability of > 22.3% are classified as high risk of developing acute refractory pain after lumbar spine surgery. This cut‐off (Index of Union) has a specificity of 76.6% (95% CI 73.2%–78.7%) and sensitivity of 53.1% (95% CI 46.9%–59.9%). The decision curve to describe the net benefit of the prediction rule versus default policy is depicted in File S4. It shows that addition of the prediction rule in clinical practice can be of benefit in preoperative risk stratification for acute refractory pain after lumbar spine surgery. To illustrate, a fictive case along with calculation of its predicted probability is described in File S4.

### 3.5 Additional Analyses

#### 3.5.1 Variable Selection for Model Development Using Multivariable Logistic Regression

The variables selected using multivariable logistic regression analysis were age, having back pain before age 45, preoperative opioid use, instrumented versus un‐instrumented surgery procedure, and anxiety were predictive factors for acute refractory pain after lumbar spine surgery. A calibration plot was obtained from this regression model, showing that the model's overall fit was good, but the range in predicted probabilities was limited (Figure 3A). The model explained 8.4% of the variation (Nagelkerke's *R* ^2^) in development of acute refractory pain, and the model's ROC‐AUC was 0.67 (95% CI 0.63–0.71) (Figure 3B).

**FIGURE 3:**
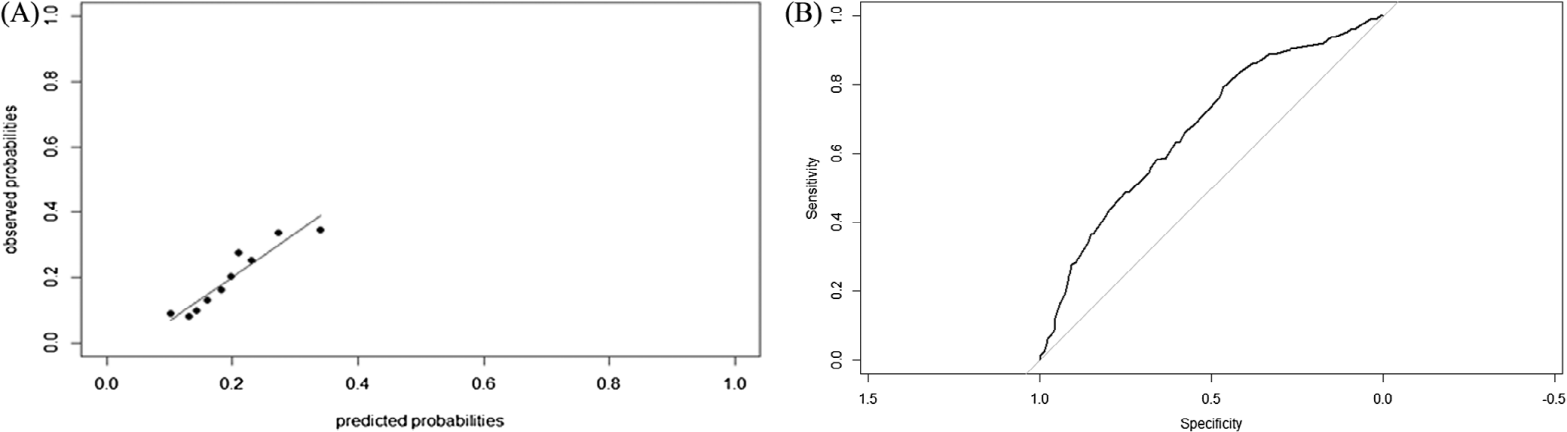
Model performance backward multivariable logistic regression. (A) Calibration plot (dotted line indicates perfect calibration); (B) ROC‐AUC 0.67 (95% CI 0.63–0.71).

#### 3.5.2 Univariable Analysis

The results of univariable analysis of the selected predictive factors are shown in File S5.

#### 3.5.3 Sensitivity Analysis

The sensitivity analysis to identify a prediction model for patients at risk for developing severe acute postoperative pain, that is, a NRS of ≥ 4 was attached in File S5.

## 4 Discussion

### 4.1 General Findings

The first aim of this study was to determine the prevalence of acute refractory pain after lumbar spine surgery. This was found to be 19.7% (*n* = 228/1156). Second, the goal was to develop and validate a clinical prediction rule for assessing preoperatively the individual risk of developing acute refractory pain. Using multivariable logistic lasso regression, the final model had performance of ROC‐AUC 0.69, 95% CI 0.66–0.73, and identified thirteen predictive factors: age, prior hospital treatment for back pain, professional sports participation, duration of back pain, back pain before age 45, numeric pain rating scale for back pain, preoperative opioid use, circumferent surgery, instrumented surgery, anxiety, catastrophizing, return‐to‐work attitude, high score for psychosocial burden of low back pain. This final model was used for the clinical prediction rule. A clinical cut‐off value was set at a predicted probability of 22.3%, enabling patient stratification into high‐ or low risk of developing postoperative acute refractory pain.

### 4.2 Comparison to Literature

The current prediction rule aligns with previous models, including that of Yang et al. [11], but there are some notable differences. In contrast to earlier studies, gender was not selected as a predictive factor in our model. Additionally, although depression is a well‐established predictor of acute refractory pain, the Hospital Anxiety and Depression Scale (HADS) could not be included due to excessive missing data. It is likely that depression would have been a relevant predictor, consistent with existing literature. Our model shares five key variables with Yang et al. [11]: age, preoperative opioid use, preoperative back pain, and type of surgery. However, we extended the model by incorporating additional factors such as previous hospital treatment for back pain, sports participation, duration of back pain, onset of back pain before age 45, anxiety, catastrophizing, return‐to‐work attitude, and psychosocial risk for persistent, disabling back pain. These variables broaden the scope of the model by addressing six additional outcome domains within the biopsychosocial framework, offering a more comprehensive approach to predicting acute refractory pain. Preoperative use of opioids (aOR 1.84, 95% CI 0.28–0.94) was the strongest predictor of acute refractory pain, which is consistent with existing research [11, 23]. Although opioids are effective for acute pain, chronic use is discouraged due to risk of addiction, tolerance or sedation [23, 24], prompting calls for preoperative opioid tapering for chronic non‐cancer pain to improve surgical outcomes [24]. Opioid tapering can reduce catastrophizing and anxiety, both predictive in our model [25]. Targeted (cognitive‐behavioral) therapies for high catastrophizing are promising, as is a person‐centered approach to tapering opioids and addressing modifiable risk factors [26, 27, 28].

Other modifiable predictive risk factors in our model included were the return‐to‐work attitude, back pain, and the psychosocial risk for persistent, disabling back pain. Professional sports participation is a strong predictor for acute refractory pain (aOR 8.04, 95% CI 0.18–3.98). Although there are few professional athletes in our study sample (*n* = 3), all are confirmed to suffer from acute refractory pain. This may relate to psychosocial factors such as fear of pain and reinjury, concerns about not returning to pre‐injury level of proficiency, mood and sleep disturbance [29, 30]. It is important to address the common comorbid conditions of depression, anxiety and sleep disorder in this specific patient group [30]. Nonmodifiable risk factors included circumferent surgery, instrumented surgery, younger age, onset and duration of back pain, and prior hospital treatment for back pain.

### 4.3 Strengths and Limitations

The methodology used in this study is the main strength. First, we used real‐world registry data of a large databank comprising 105 unique potentially predictive factors for model‐development. Registry data are considered the ‘gold standard’ and most reliable for measuring healthcare use [31]. Second, our model comprises biopsychosocial patient‐related factors, which are usable and measurable in clinical practice, offering a valuable tool for risk stratification and decision‐making and consequently provide preoperative tailored care. Third, our multivariable logistic lasso regression model revealed more relevant predictive factors compared to the additional multivariable logistic regression analysis without lasso. The latter is limited to not exceed acceptable events per variable and therefore excluded relevant predictive factors which were indeed selected by the multivariable logistic lasso regression [32]. On top of that, the lasso logistic regression performed better (ROC‐AUC of 0.69, 95% CI 0.66–0.73 for lasso logistic regression vs. 0.67, 95% CI 0.63–0.71 for logistic regression without lasso). This, along with improved generalizability and less risk of optimism, shows that multivariable logistic lasso regression is a superior method for selection of factors than a parsimonious model with multivariable logistic regression analysis. Fourth, in this study, we used a composite outcome measure based on clinical presentation, rather than relying solely on pain intensity scores like the NRS, to better capture the complexity of acute refractory postoperative pain. While NRS is commonly used, it may not fully reflect the patient's actual pain experience due to preoperative NRS and other perioperative influences [33]. Although some components of the composite outcome may be influenced by local clinical practice, the outcome was designed to reflect clinically meaningful failure of postoperative pain management rather than pain intensity alone. Our approach offers a more comprehensive assessment of the clinical and phenotypical characteristics of patients who experience acute refractory postoperative pain. To ensure comparability with existing models, we also incorporated a prediction rule for severe pain (NRS ≥ 4 at rest) during PACU stay (File S5).

Some limitations should be touched upon. First, the specific surgical indication (e.g., disc herniation, spinal stenosis, spondylolisthesis, or degenerative disc disease) was not available. Consequently, potential differences in acute refractory postoperative pain between surgical indication groups could not be explored.

Second, although the model identified clinically relevant predictors of acute refractory postoperative pain, its predictive performance was only fair (ROC‐AUC 0.69; Nagelkerke's *R* ^2^ 8.4%). Therefore, this model does not capture all factors contributing to postoperative pain and should not be considered sufficient for independent clinical decision‐making. This suggests that unmeasured factors, such as genetic or perioperative influences, may contribute to acute refractory pain [15, 34].

Third, the composite outcome measure may partly reflect local clinical practice patterns. Components such as rescue analgesic administration, anesthesiologist consultation, and prolonged PACU stay may be influenced by institutional pain management protocols and clinician behavior. Consequently, the observed prevalence of acute refractory postoperative pain and the performance of the prediction model may vary across institutions. Therefore, external validation and impact analysis are still needed to assess the model's generalizability to other populations, hospitals, and healthcare settings with potentially different prescribing practices and perioperative care pathways.

Fourth, the study included patients over a 10‐year period (2012–2022). During this timeframe, changes in surgical techniques, perioperative care pathways, and opioid prescribing practices may have occurred. As a result, temporal changes in clinical practice may have influenced both the prevalence of acute refractory postoperative pain and the performance of the prediction model.

### 4.4 Clinical Use and Future Implications

Our final prognostic model converted to a clinical prediction rule captures clinical patients with acute refractory postoperative pain, despite the use of a multimodal analgesic regimen, offering a valuable tool for risk stratification and decision‐making in lumbar spine surgery. Although model performance is fair, it improves upon default policy and supports anticipatory care for high‐risk patients, such as preoperative habituation on a multidimensional basis. Preoperative interventions such as preoperative opioid tapering programs and cognitive behavioral therapy may improve outcomes and reduce (persistent) postoperative pain [24, 35]. The tool can also support patient education, shared decision making, and managing expectations about postoperative pain, which have been proven to be of relevance in the existence of postoperative pain in previous research [36]. The present study focused exclusively on acute refractory postoperative pain and was not designed to evaluate long‐term outcomes. Future longitudinal studies are needed to investigate whether acute refractory postoperative pain is associated with the development of persistent postoperative pain and other long‐term outcomes after lumbar spine surgery. For research purposes, the identified predictive factors in this study can help correction for confounders in future pain studies into spine surgery. Future research should focus on further development of the current prediction rule in terms of generalizability and impact analysis, as well as on treatment or preoperative interventions to manipulate modifiable predictive factors. Effects of these interventions could then be evaluated by assessing impact on the prediction model and additional risk stratification. The clinical prognostic rule can then become a marker of enhancement of quality of care and be of contribution to personalized, tailored decision‐making in daily clinical practice.

## 5 Conclusion

One in five patients experience acute refractory pain after lumbar spine surgery. Our prognostic model, converted to a clinical prediction rule, can be of added value to support anesthesiologists on the preoperative screening but should not replace clinical judgment. This study emphasizes the need for tailored, person‐centered approaches to manage postoperative pain and reduce the incidence of acute refractory pain after spine surgery. A better personalized preoperative preparation program should be developed that focuses on negative phenotypical characteristics, in order to better manage postoperative pain. This in turn could lead to reduction in postoperative opioid use, recovery time, healthcare costs and improved patient satisfaction. Future research should focus on external validation, adding factors which are potentially explaining for the risk of acute refractory pain to improve accuracy of the predictions, and impact analysis should be executed to assess the clinical value of this prediction model.

## Author Contributions

I.H.W., K.C.P.V., R.L.M.B., M.L.H. contributed to the conception and design of the study. I.H.W., M.W.H. collected the data and performed the statistical analyses. I.H.W., M.W.H., K.C.P.V., R.L.M.B., M.L.H., A.R., and M.G. interpreted the data. I.H.W., K.C.P.V., R.L.M.B., M.L.H., M.W.H., A.R., and M.G. drafted the manuscript. All authors critically revised the manuscript for important intellectual content and approved the final version of the manuscript. All authors agree to be accountable for all aspects of the work.

## Funding

The authors have nothing to report.

## Ethics Statement

A waiver was obtained by the medical research ethics committee, METC Oost‐Nederland, as this study is not subject to Medical Research Involving Human Subjects Act (WMO) (reference 2023–16,799).

## Consent

This study involved the use of pseudonymized data. Preoperative data from the institutional spine registry were matched with operative identifiers to obtain postoperative outcome data from the electronic medical record. Individual patient consent was not required in accordance with institutional ethical standards.

## Conflicts of Interest

Dr. Vissers is an Editorial Board Member of Pain Practice and a co‐author of this article. To avoid any potential bias, they were not involved in the editorial review or decision‐making process regarding the acceptance of this article.

## Supporting information


**File S1:** Pilot outcome measure.

**File S2:** Potential predictive variables.

**File S3:** GitHub code sharing Rstudio.

**File S4:** Determination of cut‐off value for clinical use.

**File S5:** Univariable analysis and model with outcome measure NRS ≥ 4.

## Data Availability

The data that support the findings of this study are available on request from the corresponding author. The data are not publicly available due to privacy or ethical restrictions.
