# Supplementary material for: Development and Validation of a Clinical Prediction Rule to Identify Preoperatively Patients With Acute Refractory Pain After Lumbar Spine Surgery
Source: Pain Pract. 2026 Oct 14;26(8):e70213. doi: 10.1111/papr.70213 (PMC13669519; doi:10.1111/papr.70213)
Supplement: Supplementary file 1 [file PAPR-26-0-s001.docx]

**Supplementary file 2: potential predictive variables**

**Suppl. File 2 Table 1** Potential predictive variables, including reasons for in- or exclusion. Variables in bold are included variables in logistic regression.

| Domain | Category | Measurement | Included as predictor | Reason exclusion |
| --- | --- | --- | --- | --- |
| Socio-demographic (Personal) | **Age** | years | Yes |  |
|  | **Gender** | man = 0; woman = 1 | Yes |  |
|  | Body Mass Index | Self-reported weight/height² | No | missing values |
| Socio-demographic (Health) | **American Society of Anesthesiologists score** | range 1-5 | Yes |  |
|  | **Smoking** | no = 0; yes = 1 | Yes |  |
|  | Do you have a history of smoking? | no = 0; yes = 1 | No | no events (for outcome) |
|  | How much do you smoke? |  | No | missing values |
|  | Previous episodes of back pain | no = 0; yes = 1 | No | double variables |
|  | **Previous back surgery** | no = 0; yes = 1 | Yes |  |
|  | Amount of back surgeries |  | No | missing values |
|  | Did you have back surgery less than 6 months ago? | no = 0; yes = 1 | No | no events (for outcome) |
|  | **Interventions in the past** | no = 0; yes = 1 | Yes |  |
|  | - Pain Program in Revalidation (RealHealth) | no = 0; yes = 1 | No | double variables |
|  | - Therapy at Spineamp Joint Centre | no = 0; yes = 1 | No | double variables |
|  | - Therapy in hospital | no = 0; yes = 1 | No | double variables |
|  | - Treatment at physical therapist | no = 0; yes = 1 | No | double variables |
|  | - Treatment at psychologist | no = 0; yes = 1 | No | double variables |
|  | - Winnock Programme | no = 0; yes = 1 | No | double variables |
| Socio-demographic (Social) | Education | no education = 0; primary education = 1; LBO = 2; MBO = 3; MIM = 4; HBO = 5; HAW = 6; university = 7 | No | missing values |
|  | Social status | married = 1; living together = 2; single = 3 | No | not clinically relevant |
|  | Functioning – leisure | no = 0; yes = 1 | No | not clinically relevant |
|  | Social support | no = 0; yes = 1 | No | not clinically relevant |
|  | Sport activity | yes, recreational = 1; yes, professional = 2; no = 3 | No | not clinically relevant |
| Socio-demographic (Work) | Socio-economic status – having a job | yes, >30h/w = 0; yes, <30h/w = 1; no, looking for work = 2; no, volunteer = 3; no, stay at home parent = 4; no, retired = 5; no, sick leave = 6; no, incapacitated = 7; no, student = 8; no, different = 9 | No | no events (for outcome) |
|  | Socio-economic status – having a physically demanding job | not working = 1; light = 2; moderate = 3; heavy = 4 | No | not clinically relevant |
|  | **Work satisfaction** | no = 0; yes = 1 | Yes |  |
|  | Work functioning | yes, partially = 1; yes, complete = 2; no = 3 | No | no events (for outcome) |
|  | Sick leave | no = 0; yes = 1 | No | double variables |
|  | Sick leave in the past 6 months | no = 0; yes = 1 | No | no events (for outcome) |
|  | Receiving disability benefit | yes, complete = 1; yes, partially = 2; no = 3 | No | no events (for outcome) |
|  | Receiving pension benefit | yes, partially = 1; yes, complete = 2; no = 3 | No | not clinically relevant |
|  | **Litigation** | no = 0; yes = 1 | Yes |  |
| Pain | Back pain duration | No back pain = 1; less than 3 months = 2; 3-12 months = 3; 1-2 year = 4; more than 2 year = 5 | No | double variables |
|  | Leg pain duration | No leg pain = 1; less than 3 months = 2; 3-12 months = 3; 1-2 years = 4; more than 2 years = 5 | No | double variables |
|  | **More than 2 years back pain duration** | 2-5 years = 1; 6-10 years = 2; more than 10 years = 3; no back pain = 4 | Yes |  |
|  | **Onset of back pain before 45 years of age** | no = 0; yes = 1 | Yes |  |
|  | **Back pain intensity** | Numeric Rating Scale (score 0-10) | Yes |  |
|  | Neuropathic back pain intensity | DN4 scores 0 - 10 (neuropathy ≥ 4) | No | missing values |
|  | **Leg pain intensity** | Numeric Rating Scale (score 0-10) | Yes |  |
|  | Neuropathic leg pain intensity | DN4 scores 0 - 10 (neuropathy ≥ 4) | No | missing values |
|  | Back pain had slow onset | no = 0; yes = 1 | No | not clinically relevant |
|  | Back pain does not improve with rest | no = 0; yes = 1 | No | not clinically relevant |
|  | Back pain improves with movement | no = 0; yes = 1 | No | not clinically relevant |
|  | Back pain improves with standing up | no = 0; yes = 1 | No | not clinically relevant |
|  | Pain (SF-36) |  | No | missing values |
|  | Preoperative use of analgesics | yes, sometimes = 1; yes, regularly = 2; no = 3 | No | double variables |
|  | - **Preoperative use of anticonvulsants** | no = 0; yes = 1 | Yes |  |
|  | - **Preoperative use of benzodiazepines** | no = 0; yes = 1 | Yes |  |
|  | - Preoperative use of NSAIDs | no = 0; yes = 1 | No | not relevant in literature |
|  | - **Preoperative use of opioids** | no = 0; yes = 1 | Yes |  |
| Somatic | **Circumferent surgery** | no = 0; yes = 1 | Yes |  |
|  | **Instrumented vs uninstrumented** | uninstrumented = 0; instrumented = 1 | Yes |  |
|  | Loss of neurological function | Numbness legs/feet | No | not clinically relevant |
|  | Loss of neurological function | Numbness hips/thighs/buttocks | No | not clinically relevant |
|  | Loss of neurological function | Incontinence | No | not clinically relevant |
|  | Loss of neurological function | Loss of muscle power legs/feet | No | not clinically relevant |
|  | Loss of neurological function | Paresthesia legs/feet | No | not clinically relevant |
|  | General health experience (SF-36) |  | No | missing values |
|  | Physical functioning (SF-36) |  | No | missing values |
|  | Changes in health status (SF-36) |  | No | missing values |
| Somatic (Comorbidities) | Arthritis | no = 0; yes = 1 | No | no events (for outcome) |
|  | Asthma/COPD/CARE | no = 0; yes = 1 | No | no events (for outcome) |
|  | Central nervous system disease | no = 0; yes = 1 | No | no events (for outcome) |
|  | Consequences of stroke | no = 0; yes = 1 | No | no events (for outcome) |
|  | Depression | no = 0; yes = 1 | No | no events (for outcome) |
|  | Diabetes Mellitus | no = 0; yes = 1 | No | no events (for outcome) |
|  | Heart disease | no = 0; yes = 1 | No | no events (for outcome) |
|  | Hemiplegia/paraplegia | no = 0; yes = 1 | No | no events (for outcome) |
|  | History of arthritis | no = 0; yes = 1 | No | missing values |
|  | Hypertension | no = 0; yes = 1 | No | no events (for outcome) |
|  | Intermittent claudication | no = 0; yes = 1 | No | no events (for outcome) |
|  | Kidney disease | no = 0; yes = 1 | No | no events (for outcome) |
|  | Liver disease | no = 0; yes = 1 | No | no events (for outcome) |
|  | Other comorbidities | no = 0; yes = 1 | No | not clinically relevant |
|  | Rheumatoid arthritis | no = 0; yes = 1 | No | no events (for outcome) |
| Somatic (Red Flags) | AIDS/HIV | no = 0; yes = 1 | No | no events (for outcome) |
|  | Calin criteria for axial spondylarthritis - Bowel disease | no = 0; yes = 1 | No | no events (for outcome) |
|  | **Nightly pain** | no = 0; yes = 1 | Yes |  |
|  | Current steroid use | no = 0; yes = 1 | No | not clinically relevant |
|  | Deformities (e.g., scoliosis) | no = 0; yes = 1 | No | not clinically relevant |
|  | History of intravenous drug use | no = 0; yes = 1 | No | no events (for outcome) |
|  | Numbness of hips/thighs/buttocks | no = 0; yes = 1 | No | not clinically relevant |
|  | Pain in thoracic spine | no = 0; yes = 1 | No | no events (for outcome) |
|  | **Pain is constant and non-mechanical** | no = 0; yes = 1 | Yes |  |
|  | Pain started age <20 or >50 years | no = 0; yes = 1 | No | not clinically relevant |
|  | Paresthesia legs/feet | no = 0; yes = 1 | No | not clinically relevant |
|  | Previous history of malignancies | no = 0; yes = 1 | No | no events (for outcome) |
|  | Recent unexplained weight loss | no = 0; yes = 1 | No | no events (for outcome) |
|  | Significant trauma | no = 0; yes = 1 | No | not relevant in literature |
| Psychological (Psychic affect) | Did you feel down during the last few weeks? | Yes, a little = 1; yes, a lot = 2; no = 3 | No | missing values |
|  | Hospital Anxiety and Depression Scale | Depression Scale (score 0-21) | No | missing values |
|  | **Did you feel anxious during the last few weeks?** | Yes, a little = 1; yes, a lot = 2; no = 3 | Yes |  |
|  | Hospital Anxiety and Depression Scale | Anxiety scale (score 0-21) | No | missing values |
|  | Mental health status (SF-36) |  | No | missing values |
| Psychological (Cognition) | **Somatization** | My body is telling me I have something dangerously wrong: disagree = 0; agree = 1 | Yes |  |
| Psychological (Behavior) | **Expectations - outcome interventions (treatment expectations)** | Do you expect to be free of complaints after treatment? no = 0; yes = 1 | Yes |  |
|  | **Expectations - return to work** | Do you expect to be able to return to work? currently working = 1; full-time return = 2; part-time return = 3; change of job = 4; paid sick leave = 5; pension = 6 | Yes |  |
|  | Roll restriction due to emotional problems (SF-36) |  | No | missing values |
| Functioning & Quality of life | **Oswestry Disability Index** |  | Yes |  |
|  | **EQ-5D-5L total score** |  | Yes |  |
|  | **EQ-VAS** |  | Yes |  |
|  | **STarT Back Screening Tool - risk of development of persistent invalidating low back pain** | low risk = 0; medium risk = 1; high risk = 2 | Yes |  |
|  | Roll restriction due to physical problems (SF-36) |  | No | missing values |
|  | Social functioning (SF-36) |  | No | missing values |
|  | Vitality score (SF-36) |  | No | missing values |

**Suppl. File 2 Fig. 1** Flow chart of selection of predictive variables for the lasso regression
and multivariable logistic regression analysis
