# Supplementary material for: Development and Validation of a Clinical Prediction Rule to Identify Preoperatively Patients With Acute Refractory Pain After Lumbar Spine Surgery
Source: Pain Pract. 2026 Oct 14;26(8):e70213. doi: 10.1111/papr.70213 (PMC13669519; doi:10.1111/papr.70213)
Supplement: Supplementary file 2 [file PAPR-26-0-s002.docx]

**Supplementary file 3: GitHub code sharing Rstudio**

library(rms)

library(foreign)

library(pROC)

library(ResourceSelection)

library(psfmi)

library(purrr)

library(stringr)

library(tibble)

library(dplyr)

library(ggplot2)

library(splines)

library(readxl)

library(rlang)

library(broom)

library(tidyr)

library(glmnet)

library(DHARMa)

library(gap)

library(car)

library(dcurves)

library(tidyverse)

#1. link entire dataset to object dat

dat <- read_excel('PRE-OPT-PAIN_final_23102023.xlsx')

dd <- datadist(dat)

options(datadist='dd')

view(dat)

summary(dat)

#1.1 define Variable as categorical

dat$Meerdan2jaarrugpijn <- factor(dat$Meerdan2jaarrugpijn)

dat$Gespannenofangstigafg.week <- factor(dat$Gespannenofangstigafg.week)

dat$Pijnstillersvoorrugklachten <- factor(dat$Pijnstillersvoorrugklachten)

dat$Terugkeernaarwerkmogelijk <- factor(dat$Terugkeernaarwerkmogelijk)

dat$SBST_cat <- factor(dat$SBST_cat)

dat$Hoelanguitstralendepijnbeen <- factor(dat$Hoelanguitstralendepijnbeen)

dat$Heeftueenbetaaldebaan <- factor(dat$Heeftueenbetaaldebaan)

dat$Werkuitvoerenondanksklachten <- factor(dat$Werkuitvoerenondanksklachten)

dat$Hoelanghuidigerugklachten <- factor(dat$Hoelanghuidigerugklachten)

dat$OntvangtuAOWpensioen <- factor(dat$OntvangtuAOWpensioen)

dat$OntvangtueenWAOuitkering <- factor(dat$OntvangtueenWAOuitkering)

dat$Hoezwaarisuwhuidigewerk <- factor(dat$Hoezwaarisuwhuidigewerk)

dat$Burgelijkestatus <- factor(dat$Burgelijkestatus)

dat$Sportu <- factor(dat$Sportu)

dat$ASAscore <- factor(dat$ASAscore)

#2. testing assumptions

#2.1 linear assumption (restricted cubic spline regression)

#Continuous variables are NPRS_back, ODI_preop, EQ5D_total, Age, EQVAS, DN4_back, NPRS_leg

#Linear regression analysis including spline basis functions xt1, xt2 and xt3

lrm(NR ~ rcs(Age, 3), data = dat)

lrm(NR ~ rcs(NPRS_back, 3), data = dat)

lrm(NR ~ rcs(ODI_preop, 3), data = dat)

lrm(NR ~ rcs(EQ5D_total, 3), data = dat)

lrm(NR ~ rcs(EQVAS, 3), data = dat)

#How to implement spline variable in model:

test <- lrm(NR ~ rcs(Age, quantile(Age, c(0.1, 0.5, 0.9))) + SBST_cat, data=dat)

test

#2.2 Data inspection: are enough data points present for catagorical variables? Making crosstabs:

table(x = dat$Meerdan2jaarrugpijn, y = dat$NR)

table(x = dat$Gespannenofangstigafg.week, y = dat$NR)

table(x = dat$Pijnstillersvoorrugklachten, y = dat$NR)

table(x = dat$Terugkeernaarwerkmogelijk, y = dat$NR)

table(x = dat$SBST_cat, y = dat$NR)

table(x = dat$Hoelanguitstralendepijnbeen, y = dat$NR)

table(x = dat$Heeftueenbetaaldebaan, y = dat$NR)

table(x = dat$Werkuitvoerenondanksklachten, y = dat$NR)

table(x = dat$Hoelanghuidigerugklachten, y = dat$NR)

table(x = dat$EducatieCAT, y = dat$NR)

table(x = dat$OntvangtuAOWpensioen, y = dat$NR)

table(x = dat$OntvangtueenWAOuitkering, y = dat$NR)

table(x = dat$Hoezwaarisuwhuidigewerk, y = dat$NR)

table(x = dat$Burgelijkestatus, y = dat$NR)

table(x = dat$Sportu, y = dat$NR)

table(x = dat$Somberafgelopenweek, y = dat$NR)

table(x = dat$ASAscore, y = dat$NR)

table(x = dat$Eerdereoperatieaanrug, y = dat$NR)

table(x = dat$Eerdertherapiejanee, y = dat$NR)

table(x = dat$Therapiepijnprogrammainrevalidatiecentrum, y = dat$NR)

table(x = dat$Therapieziekenhuis, y = dat$NR)

table(x = dat$Behandelingbijfysiotherapeut, y = dat$NR)

table(x = dat$Behandelingbijpsycholoog, y = dat$NR)

#2.3 Evaluating multicollinearity with VIF test. Cut-off > 10

model <- lrm(NR ~ Age + ASAscore + Circumferent_dichotomous + Eerdereoperatieaanrug + Eerdertherapiejanee + EQ5D_total

+ EQVAS + Geenrugklachtennabehandeling + Gespannenofangstigafg.week + Lichaamgevaarlijkdich + Meerdan2jaarrugpijn

+ NPRS_back + NPRS_leg + ODI_preop + Preopantiepilepticsuse + Preopbenzouse + Preopopioiduse + RF_nachtelijkepijn

+ RF_NietMechanisc + Rugvoor45e + SBST_cat + Sex + Smoking + Terugkeernaarwerkmogelijk + Tevredenmethuidigewerk

+ Treatmentcode_dich + Verwikkeldinrechtzaak, data=dat)

vif(model)

#3. Lasso regression

#3.1 Model development Lasso

X <- model.matrix(NR ~ ., data = dat)[, -1]

Y <- dat$NR

fit.lasso <- glmnet(y=Y, x=X, nlambda=100, alpha=1, family="binomial")

plot(fit.lasso, xvar="lambda", label=TRUE)

coef(fit.lasso)

legend("topright", lwd = 1, col = 1:6, legend = FALSE, cex = .7)

cv.lasso <- cv.glmnet(x=X, y=Y, alpha = 1, family = "binomial", nfolds = 10)

plot(cv.lasso)

cv.lasso$lambda.min

coef(cv.lasso, cv.lasso$lambda.min)

cv.lasso$lambda.1se

coef(cv.lasso, cv.lasso$lambda.1se)

fitlas <- glmnet(y=Y, x=X, alpha=1, lambda=cv.lasso$lambda.min, family="binomial")

fitlas

coef(fitlas)

predlas <- predict(fitlas, newx=X, type="response")

predlas

predictionrule <- lrm(NR ~ Circumferent_dichotomous + Age + Treatmentcode_dich + Preopopioiduse + SBST_cat + NPRS_back + Terugkeernaarwerkmogelijk + Meerdan2jaarrugpijn + Sportu + Gespannenofangstigafg.week + Rugvoor45e + Lichaamgevaarlijkdich + Therapieziekenhuis, x=TRUE, y=TRUE, data = dat)

predictionrule

#3.2 Model performance Lasso

roc <- pROC::roc(Y, as.numeric(predlas), ci=TRUE, plot=TRUE)

#find the best threshold

coords(roc, "best", ret="all", transpose=FALSE)

#obtain confidence intervals of se and sp

ci.thresholds(roc, threshold=0.2225553, conf.level=0.95, stratified=FALSE)

coords(roc, 0.30, ret="all", transpose=FALSE)

plot(specificity + sensitivity ~ threshold, coords(roc, "all", transpose=FALSE),

type="l", log="x", subset = is.finite(threshold))

plot(precision ~ recall,

coords(roc, "all", ret = c("recall", "precision"), transpose = FALSE),

type="l", ylim = c(0, 100))

plot(tpr ~ fpr,

coords(roc, "all", ret = c("tpr", "fpr"), transpose = FALSE),

type="l")

#Obtain calibration curve

#Make groups based on the predicted probabilities

group.cut.las <- quantile(predlas, c(seq(0, 1, 0.1)))

group.las <- cut(predlas, group.cut.las)

pred.prob.las <- tapply(predlas, group.las, mean)

#Observed probabilities

obs.prob.las <- tapply(predictionrule$y, group.las, mean)

#Calibration plot

windows()

plot(1, type="n", xlim=c(0,1), ylim=c(0,1), xlab="predicted probabilities", ylab="observed probabilities")

lines(c(seq(0, 1, 0.1)), c(seq(0, 1, 0.1)), lty=2)

points(pred.prob.las, obs.prob.las, pch=16)

library(splines)

#straight calibration line

lines(pred.prob.las, fitted(lm(obs.prob.las ~ pred.prob.las)))

#curved line

lines(pred.prob.las, fitted(lm(obs.prob.las ~ ns(pred.prob.las, df=3))))

#3.3 Clinical application

#3.3.1 Boxplot

LPlas <- predictionrule$linear.predictors

boxplot(plogis(LPlas)~predictionrule$y,)

#3.3.2. Decision curve

phat_NR <- exp(LPlas) / (1+exp(LPlas))

#3.3.3 Run the decision curve

dca(NR ~ phat_NR, data = dat, thresholds = seq(0, 0.35, 0.01), label = list(phat_NR = "Refractory Pain Model")

) %>%

plot(smooth = TRUE)

#4. Multivariable logistic regression analysis

#4.1 Model development using n=26 predictive variables (parsimonious model)

fit.large <- lrm(NR ~ Age + ASAscore + Circumferent_dichotomous + Eerdereoperatieaanrug + Eerdertherapiejanee + EQ5D_total

+ EQVAS + Geenrugklachtennabehandeling + Gespannenofangstigafg.week + Lichaamgevaarlijkdich + Meerdan2jaarrugpijn

+ NPRS_back + NPRS_leg + ODI_preop + Preopantiepilepticsuse + Preopbenzouse + Preopopioiduse + RF_nachtelijkepijn

+ RF_NietMechanisc + Rugvoor45e + SBST_cat + Sex + Smoking + Terugkeernaarwerkmogelijk + Treatmentcode_dich

+ Verwikkeldinrechtzaak, x=TRUE, y=TRUE, data = dat)

fit.large

validate(fit.large, B=250, bw=T, rule="p", type="individual", sls=0.05)

#4.2 Model adjustment

#Shrinkage of coefficients

fit <- lrm(NR ~ Age + Preopopioiduse + Rugvoor45e + Treatmentcode_dich + Gespannenofangstigafg.week, x = T, y = T, data = dat)

coef.new <- coef(fit)[-1]*0.8716 #Use the regression coefficients of the development data =coef(fit) and multiply with the predictor values in the validate dataset for each person

coef.new

lp.new <- fit$x%*%coef.new

#Adjustment of intercept

intercept.new <- glm(dat$NR ~ offset(lp.new), family=binomial)

intercept.new

#Obtaining prediction rule

X <- data.frame(fit$x)

lp_final = -1.177 + (0.56743812 * X$Preopopioiduse) + (0.40452055*X$Treatmentcode_dich) + (-0.48114140 * X$Rugvoor45e) + (-0.10739366*X$Gespannenofangstigafg.week.1) + (0.65219460*X$Gespannenofangstigafg.week.2) + (-0.01103461 * X$Age)

fit.final <- lrm(dat$NR ~ lp_final, x=T, y=T)

fit.final

prob.final <- 1/(1+exp(-lp_final))

#4.3 Performance logistic regression model

\# ROC curve

ROC.final <- roc(fit.final$y, prob.final, ci=T, plot=T)

ROC.final

\# calibration plot

group.cut.final <- quantile(prob.final, c(seq(0, 1, 0.1)))

group.final <- cut(prob.final, group.cut.final)

pred.prob.final <- tapply(prob.final, group.final, mean)

obs.prob.final <- tapply(fit.final$y, group.final, mean)

windows()

plot(1, type="n", xlim=c(0,1), ylim=c(0,1), xlab="predicted probabilities", ylab="observed probabilities")

lines(c(seq(0, 1, 0.1)), c(seq(0, 1, 0.1)), lty=2)

points(pred.prob.final, obs.prob.final, pch=16) #plot the observed and predicted variabilities and assign axis limits and axis names

lines(pred.prob.final, fitted(lm(obs.prob.final ~ pred.prob.final))) #draw a straight line through the observed and predicted points

lines(pred.prob.final, fitted(lm(obs.prob.final ~ ns(pred.prob.final, df=3)))) #optional; draw a smoothed (curved) line through the points

#5. Clinical application

#5.1 Boxplot

LPb <- fit.final$linear.predictors

boxplot(plogis(LPb)~fit.final$y,)

#5.2. Decision curve

phat_NR <- exp(lp_final) / (1+exp(lp_final))

\# Run the decision curve

dca(NR ~ phat_NR, data = dat, thresholds = seq(0, 0.35, 0.01), label = list(phat_NR = "NR Model")

) %>%

plot(smooth = TRUE)

#Apply cut-off to data

dat %>% mutate(prob.final = (prob.final < 0.40 =0), (prob.final > 0.40 = 1))
