# Supplementary material for: Development and Validation of a Clinical Prediction Rule to Identify Preoperatively Patients With Acute Refractory Pain After Lumbar Spine Surgery
Source: Pain Pract. 2026 Oct 14;26(8):e70213. doi: 10.1111/papr.70213 (PMC13669519; doi:10.1111/papr.70213)
Supplement: Supplementary file 3 [file PAPR-26-0-s003.docx]

**Supplementary file 1: pilot outcome measure**

*Pilot outcome measure of acute refractory pain*

All analyses were conducted in IBM SPSS Statistics (version 29). The Syntax and relevant output is added to this supplementary material.

Before choosing the primary outcome as used in the research article, a pilot was conducted for feasibility. This pilot included patient outcome data from 2019 and included data of 380 patients having undergone spine surgery at the Sint Maartenskliniek.

At first, we glanced at the Numeric Rating Scale (NRS) at rest and in action during stay at the Post-Anesthesia Care Unit to see what is the most relevant.

DESCRIPTIVES VARIABLES=MaxNRSrest MaxNRSaction

/STATISTICS=MEAN STDDEV MIN MAX.

There was no difference in mean maximum NRS at rest and during action. Furthermore, more missing values were present for NRS in action. Therefore, further analysis was performed using the maximum NRS at rest during stay at the Post-Anesthesia Care Unit (PACU). An NRS cut-off of 7 and higher was used as first criterium for being a non-responder. The incidence of patients with an NRS ≥ 7 was as follows:

RECODE MaxNRSrest (999=SYSMIS) (7 thru Highest=1) (ELSE=0) INTO NRS7restPACU.

VARIABLE LABELS NRS7restPACU 'NRS7restPACU'.

EXECUTE.

FREQUENCIES VARIABLES=NRS7restPACU

/ORDER=ANALYSIS.

This primary criterium was recoded into three categories so that eventual calculation of non-response would be able to identify patients with all additional criteria but not having the primary criterium:

RECODE MaxNRSrest (999=999) (4 thru 6=1) (7 thru Highest=3) (Lowest thru 3=0) INTO NRS47.

VARIABLE LABELS NRS47 'NRS47'.

EXECUTE.

FREQUENCIES VARIABLES=NRS47

/ORDER=ANALYSIS.

The additional criteria were also coded:

1. Consultation of anesthesiologist

RECODE ConsultAnaesthetist (999=999) (2=0) (1=1) INTO Anaesthetistyesno.

VARIABLE LABELS Anaesthetistyesno 'Anaesthetistyesno'.

EXECUTE.

2. Action undertaken after consultation of anesthesiologist: administering clonidine, esketamine or benzodiazepines (diazepam in the Sint Maartenskliniek). These events were prevalent as follows and recoded into action undertaken yes/no.
   a. Clonidine: n = 34
   b. Ketanest: n = 17
   c. Diazepam: n = 9

RECODE ActionNRS (999=999) (1=1) (ELSE=0) INTO ActionNRSyesno.

VARIABLE LABELS ActionNRSyesno 'ActionNRSyesno'.

EXECUTE.

3. The existence of apneas, indicating much opioids needed for achieving acceptable pain levels. 4 events in the pilot group. These events were recoded into yes/no.

RECODE Apneas (999=999) (1=1) (ELSE=0) INTO Apneayesno.

VARIABLE LABELS Apneayesno.

EXECUTE.

4. Indication to stay overnight/longer on the PACU because of pain or apneas. This was the case in 24 patients from the pilot group. The variable was recoded.

RECODE PACUIndication (999=999) (1=1) (ELSE=0) INTO PACUforpain.

VARIABLE LABELS PACUforpain.

EXECUTE.

Afterwards, the composite outcome measure could be computed:

COMPUTE Nonresponder=NRS47+(Anaesthetistyesno+ActionNRSyesno+Apneayesno+PACUforpain)/4.

EXECUTE.

Incidence of non-responders could be calculated:

FREQUENCIES VARIABLES=Nonresponder

/ORDER=ANALYSIS.

We established a cut-off of 3.25 in order for a patient to be labelled as non-response. This means that the patient should have an NRS ≥ 7 AND at least 1 of the additional criteria. This resulted in an incidence of 20.7% (75/363).

The last step was to dichotomize this composite outcome measure to 0 = responder and 1 = non-responder.

RECODE Nonresponder (MISSING=999) (3.25 thru Highest=1) (ELSE=0) INTO Nonresponderdef.

EXECUTE.

FREQUENCIES VARIABLES=Nonresponderdef

/ORDER=ANALYSIS.
