# Supplementary material for: Development and Validation of a Clinical Prediction Rule to Identify Preoperatively Patients With Acute Refractory Pain After Lumbar Spine Surgery
Source: Pain Pract. 2026 Oct 14;26(8):e70213. doi: 10.1111/papr.70213 (PMC13669519; doi:10.1111/papr.70213)
Supplement: Supplementary file 4 [file PAPR-26-0-s004.docx]

**Supplementary file 5: Univariable analysis and model with outcome measure NRS ≥ 4**

**Suppl. File 5 Table 1:** Results of Univariable Analysis for Variables Associated with Refractory Pain after Lumbar Spine Surgery.

| Domain | Parameter | β | OR | 95%CI | P |
| --- | --- | --- | --- | --- | --- |
| Socio-demographic (Personal) | Age (years) | -0.02 | 0.98 | 0.97-0.99 | <0.01* |
| Socio-demographic (Health) | Previous hospital treatment for back pain  (No = 0; Yes = 1) | 0.43 | 1.54 | 1.01-2.35 | 0.045* |
| Socio-demographic (Social) | Sports participation   - No (0) - Professional (1) - Recreational (2) | 1.77  -0.08 | Reference  5.90  0.93 | Reference  0.97-35.84  0.68-1.25 | 0.12  0.05*  0.62 |
| Pain | Duration back pain   - No back pain (0) - 2-5 years (1) - 5-10 years (2) - >10 years (3) | 0.42  0.23  -0.38 | Reference  1.53  1.26  0.68 | Reference  0.99-2.35  0.88-1.79  0.42-1.11 | 0.02*  0.05*  0.21  0.12 |
|  | Back pain before age 45  (No = 0; Yes = 1) | -0.61 | 0.54 | 0.38-0.76 | <0.01* |
|  | Numeric Pain Rating Scale back pain (0-10) | 0.04 | 1.13 | 1.05-1.23 | <0.01* |
|  | Preoperative opioid use  (No = 0; Yes = 1) | 0.68 | 1.98 | 1.47-2.67 | <0.01* |
| Somatic | Circumferent surgery  (No = 0; Yes = 1) | 0.56 | 1.75 | 1.21-2.52 | <0.01* |
|  | Surgery type (Un-instrumented = 0; Instrumented = 1) | 0.54 | 1.72 | 1.25-2.36 | <0.01* |
| Psychological (Psychic affect) | Anxiety   - No (0) - Moderate (1) - Very bad (2) | -0.03  0.86 | Reference  0.97  2.36 | Reference  0.70-1.36  1.50-3.71 | <0.01*  0.87  <0.01* |
| Psychological (Cognition) | Catastrophizing  (No = 0; Yes = 1) | 0.38 | 1.46 | 1.07-2.00 | 0.02* |
| Psychological (Behavior) | Return-to-work attitude   - Currently working (0) - Fulltime return (1) - Parttime return (2) - Wish to change job (3) - Wish to keep paid sick leave (4) - Wish to keep retirement benefit (5) | -0.36  0.10  0.65  0.23  -0.33 | Reference  0.70  1.10  1.91  1.26  0.72 | Reference  0.41-1.19  0.65-1.88  1.08-3.38  0.75-2.10  0.49-1.05 | <0.01*  0.19  0.72  0.03*  0.39  0.09* |
| Functioning and quality of life | STarT Back Screening Tool^22^   - Low risk (0) - Medium risk (1) - High risk (2) | 0.41  0.85 | Reference  1.50  2.34 | Reference  0.96-2.35  1.33-4.12 | 0.01*  0.08*  <0.01* |

β =regression coefficient; OR = Odds Ratio, values of univariable regression analysis. OR>1 means a higher risk of refractory pain and an OR<1 means a lower risk of refractory pain; 95%CI = 95%Confidence Intervals for the ORs. *Statistically significant with p-values <α=0.05.

**Sensitivity analysis**

**Suppl. File 5 Table 2: Lasso regression for prediction of outcome measure NRS ≥ 4**

| Domain | Parameter | β | aOR | 95%CI | *P* |
| --- | --- | --- | --- | --- | --- |
| Socio-demographic (Personal) | Age (years) | -0.01 | 0.99 | 0.98; 1.00 | 0.10 |
| Pain | Duration back pain   - No back pain (0) - 2-5 years (1) - 5-10 years (2) - >10 years (3) | -0.09  -0.05  -0.49 | Reference  0.91  0.95  0.61 | Reference  0.53; 1.29  0.65; 1.25  0.25; 0.97 | 0.62  0.75  <0.01* |
|  | Preoperative opioid use  No = 0; Yes = 1 | 0.56 | 1.74 | 1.48; 2.00 | <0.01* |
|  | Preoperative NSAID use  No = 0; Yes = 1 | -0.39 | 0.68 | 0.39; 0.97 | <0.01* |
| Somatic | Circumferent surgery  No = 0; Yes = 1 | 0.47 | 1.59 | 1.22; 1.96 | 0.01* |
|  | Surgery type  Uninstrumented = 0; Instrumented = 1 | 0.35 | 1.41 | 1.14; 1.68 | 0.01* |
| Psychological (Psychic affect) | Anxiety   - No (0) - Yes, moderate (1) - Yes, very bad (2) | 0.40  0.05 | Reference  1.48  1.05 | Reference  0.99; 1.97  0.78; 1.32 | 0.11  0.74 |
| Functioning and Quality of Life | EQ5D total score | -0.44 | 0.65 | 0.20; 1.10 | 0.06 |

β =regression coefficient; OR = Odds Ratio, values of univariable regression analysis. OR>1 means a higher risk of refractory pain and an OR<1 means a lower risk of refractory pain; 95%CI = 95%Confidence Intervals for the ORs. *Statistically significant with p-values <α=0.05.

**Suppl. File 5 Figure 1: ROC-AUC Lasso regression for prediction of outcome measure NRS ≥ 4.**
ROC-AUC was found to be 0.65 (0.62-0.68), Nagelkerke’s R² 0.09.
