# Supplementary material for: Development and Validation of a Clinical Prediction Rule to Identify Preoperatively Patients With Acute Refractory Pain After Lumbar Spine Surgery
Source: Pain Pract. 2026 Oct 14;26(8):e70213. doi: 10.1111/papr.70213 (PMC13669519; doi:10.1111/papr.70213)
Supplement: Supplementary file 5 [file PAPR-26-0-s005.docx]

**Supplementary file 4 – determination of cut-off value for clinical use**

**Clinical prediction rule**

A cut-off for clinical use was determined using the Index of Union. This is the “optimal” cut-point which classified the most of the individuals correctly and thus least of them incorrectly^21^. This resulted in a cut-off in probability of 22.3%, with an accompanying specificity of 76.6% (95% CI 73.2-78.7%) and sensitivity of 53.1% (95% CI 46.9-59.9%).

**Suppl. File 4 Table 1:** Cut-off point determined through the Index of Union, associated observed and predicted risk score probabilities of refractory pain and test characteristics of prediction rule (%)

| Threshold probability | Sensitivity | Specificity (95% CI) | Accuracy | PPV | NPV | TP | TN | FP | FN |
| --- | --- | --- | --- | --- | --- | --- | --- | --- | --- |
| **22.3%** | **53.1% (46.9-59.9)** | **76.6% (73.2-78.7)** | **71.5%** | **35.2%** | **86.9%** | **118** | **688** | **217** | **104** |

Abbreviations: PPV = positive predictive value; NPV = negative predictive value; TP = true positives; TN = true negatives; FP = false positives; FN = false negatives

To illustrate, the following example of a fictive case is described:

$$y= -1.9428$$

$$-0.01\cdot(54) \left( age\left( years \right) \right)$$

$$+0.41\cdot(1) \left( previous hospital treatment for back pain:yes=1 \right)$$

$$+2.08\cdot\left( 0 \right) \left( sports participation:no \right)$$

$$-0.35\cdot\left( 1 \right) \left( duration of back pain:>10 years \right)$$

$$-0.54\cdot(1) \left( back pain before age 45:yes=1 \right)$$

$$+0.04\cdot\left( 7 \right) \left( Numeric Pain Rating Scale for back pain\left( 0-10 \right)=7 \right)$$

$$+0.61\cdot(1) \left( preoperative opioid use:yes=1 \right)$$

$$+0.36\cdot(1) \left( circumferent surgery:yes=1 \right)$$

$$+0.33\cdot(1) \left( surgery type: instrumented=1 \right)$$

$$-0.22\cdot\left( 0 \right) \left( anxiety:no \right)$$

$$+0.29\cdot\left( 0 \right) \left( catastrophizing:no=0 \right)$$

$$+0.36\cdot\left( 1 \right) \left( return-to-work attitude:wish to change job \right)$$

$$+0.20\cdot(1) \left( STarT Back Screening Tool:medium risk=1 \right)$$

$$y=-1.9428+0.36-0.54+0.33+0.41+0.61-0.54+0.20+0.28-0.35+0.36=-0.7498$$

The probability $\left( p \right)$is $\frac{1}{1+e^{0.7498}} \approx0.3209$. So the chance of the patient in this example of developing refractory pain is 32.1%. The cut-off value for stratification into high or low risk was 22.3%, which means that the patient in this example would be stratified in the high risk category of developing postoperative refractory pain.

**(A)	(B)**

**(C)	(D)**

**Suppl. File 4 Figure 1: Determination of best cut-off value for clinical use.** A = boxplot for manageable pain (0) and refractory pain (1); B = a sensitivity + specificity vs. cut-off diagram; C = ROC-curve with TPR and FPR instead of SE/SP; D = Decision curve to describe the net benefit of the prediction model vs default policy. Green line = treat none; red line = treat all; blue line = treatment according to prediction model. Abbreviations: TPR = true positive rate, FPR = false positive rate, SE = sensitivity, SP = specificity.
